# Tooth X-Ray Image Segmentation Based on ResU-Net with Coordinate Attention and Boundary-Aware Mechanisms

**DOI:** 10.3390/s26123880

**Published:** 2026-06-18

**Authors:** Jie Xiong, Qiong Lou, Fang Lu

**Affiliations:** School of Science, Zhejiang University of Science and Technology, No. 318 Liuhe Road, Hangzhou 310023, China; 222409252009@zust.edu.cn (J.X.); louqiong@zust.edu.cn (Q.L.)

**Keywords:** ResU-Net, coordinate attention, boundary-aware module, panoramic dental X-ray segmentation

## Abstract

Accurate tooth segmentation plays a crucial role in computer-aided dental diagnosis and treatment planning, particularly in applications such as tooth detection, lesion localization, orthodontic analysis, and implant surgery. However, panoramic dental X-ray images often suffer from tooth adhesion, low contrast, and blurred boundaries, making precise delineation difficult and potentially compromising downstream clinical analysis. To address these challenges, we propose a boundary-aware segmentation framework, termed Boundary-Aware ResU-Net (BA-ResUNet), which is built upon a ResU-Net backbone and enhanced with Coordinate Attention (CA) and explicit boundary modeling mechanisms. Specifically, CA modules are introduced into the encoder to improve spatial representation and positional awareness. In addition, a Boundary Extraction Module (BEM) is designed to capture boundary priors from shallow and deep features, while a Boundary Injection Module (BIM) progressively incorporates these cues into the decoder through foreground enhancement and background suppression. This design enables the network to better preserve inter-tooth gaps and improve boundary delineation. Experiments on the MICCAI STS-2D dental dataset demonstrate that the proposed method achieves superior performance in terms of Dice and IoU compared with representative existing methods. Ablation and qualitative analyses further show that CA and BEM/BIM play synergistic roles in improving regional overlap and boundary localization, particularly in challenging cases involving adhesion, low contrast, and indistinct contours. These results indicate that the proposed framework provides a reliable and effective solution for panoramic tooth segmentation and has promising potential for computer-aided dental applications.

## 1. Introduction

Oral health is essential to overall human health. Dental diseases such as caries, periodontitis, and malocclusion not only affect chewing function and appearance, but may also be associated with systemic diseases [1]. In recent decades, digital dentistry has advanced rapidly, with dental X-ray imaging, especially panoramic radiographs, becoming important for clinical diagnosis and treatment planning [2]. Panoramic images capture the entire dental arch and jaw structure in a single exposure, providing crucial information for tooth localization, impacted tooth detection, implant planning, and orthodontic design. Therefore, accurate tooth segmentation from panoramic X-ray images is a prerequisite for intelligent computer-aided dental diagnosis systems, as it can significantly improve diagnostic efficiency and provide quantitative support for lesion analysis, root assessment, and personalized treatment planning [3,4].

**Figure 1:**
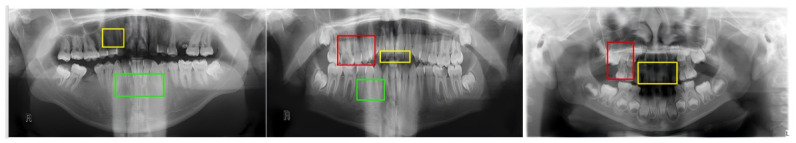
Challenges in accurate tooth segmentation from panoramic dental X-ray images. Red, yellow, and green boxes indicate boundary adhesion, low contrast, and blurred boundaries, respectively, posing challenges to tooth delineation.

Despite its clinical value, automatic tooth segmentation in dental X-ray images remains challenging due to several inherent image quality issues. Specifically, the grayscale intensity between teeth and surrounding alveolar bone is often similar, resulting in overall low contrast. Meanwhile, the inter-tooth gaps are narrow and susceptible to noise interference, making adjacent teeth prone to adhesion during segmentation [5]. Moreover, tooth boundaries frequently appear blurred or indistinct, further complicating accurate delineation and boundary localization. Importantly, these difficulties rarely occur in isolation. As shown in Figure 1, the red-boxed regions indicate boundary adhesion between adjacent teeth, where narrow inter-tooth gaps and overlapping grayscale responses make neighboring teeth difficult to separate. The yellow-boxed regions correspond to low-contrast areas, in which the grayscale difference between teeth and surrounding tissues is weak, causing tooth structures to blend into the background. The green-boxed regions highlight blurred boundaries, where tooth contours are indistinct and boundary localization becomes unstable. These challenges often coexist within the same panoramic dental X-ray images, collectively increasing the difficulty of accurate tooth segmentation.

In recent years, deep learning techniques have achieved remarkable progress in medical image analysis [6]. Unlike traditional handcrafted feature-based methods, deep learning models can automatically learn multi-scale representations through end-to-end training, showing strong adaptability in complex structure recognition. In medical image segmentation, the encoder–decoder architecture represented by U-Net [7] has become the mainstream framework, where skip connections effectively fuse shallow spatial information with deep semantic features. Variants such as ResU-Net [8] and U-Net++ [9] further enhance feature representation by introducing residual connections or nested skip pathways. In particular, ResU-Net alleviates the vanishing gradient problem in deep networks through residual learning and improves feature propagation, making it a robust backbone for medical segmentation tasks.

Nevertheless, standard convolutional neural networks (CNNs) are inherently limited by their local receptive fields. Transformer-based segmentation architectures have been introduced to improve long-range dependency modeling [10,11]; however, the main difficulty in panoramic dental X-ray segmentation lies in fine-grained boundary ambiguity, narrow inter-tooth gaps, and foreground over-expansion in low-contrast regions. Therefore, instead of directly replacing the backbone with a more complex Transformer architecture, this study adopts a stable ResU-Net framework and focuses on coordinate-aware spatial enhancement and explicit boundary-guided decoding.

To address the above challenges, we propose a boundary-aware tooth segmentation method based on the ResU-Net backbone. First, ResU-Net is adopted as the backbone to provide stable feature representation through residual connections. Second, a CA module [12] is introduced into the encoder to enhance spatial awareness by encoding positional information into channel attention. Furthermore, a BEM and BIM [13] are designed to explicitly model tooth boundaries. During decoding, boundary priors are injected via foreground enhancement and background suppression, effectively alleviating tooth adhesion and improving boundary precision.

In summary, the main contributions of this paper are as follows:

1. We propose a task-oriented BA-ResUNet framework specifically designed for panoramic dental X-ray segmentation under challenging conditions, including tooth adhesion, blurred boundaries, and low contrast.
2. To the best of our knowledge, this study is the first to develop a unified coordinate- and boundary-aware ResU-Net framework for dental X-ray segmentation. Specifically, CA is embedded into the encoder to capture direction-aware positional information and the global spatial arrangement of teeth. BEM extracts explicit boundary priors from multi-level shallow and deep features, while BIM progressively injects these priors into the decoder through boundary-guided foreground enhancement and background suppression. This design helps reduce adjacent-tooth adhesion, suppress foreground over-expansion, and preserve narrow inter-tooth gaps.
3. Extensive experiments, comparative analyses, ablation studies, and visualization results on the public datasets demonstrate that the proposed framework has clear advantages in improving regional overlap, boundary localization accuracy, and adjacent-tooth separation performance.

## 2. Related Work

### 2.1. Dental X-Ray Segmentation Methods

In recent years, tooth segmentation in panoramic dental X-ray images has attracted increasing attention, especially with the development of the MICCAI STS-2D tooth segmentation benchmark [14]. Based on recent methods proposed for this benchmark and related studies, existing approaches can be roughly divided into several categories according to their technical strategies [14,15,16,17,18,19,20,21].

First, CNN-based and multi-scale feature fusion methods aim to improve region-level representation. Shen et al. [15] proposed VCMix-Net+, which adopts a half-image strategy and VCMix-based augmentation to enhance segmentation robustness. Wang et al. [16] introduced a CNN-based multi-scale semantic segmentation method for two-dimensional panoramic X-ray images, improving tooth segmentation by extracting features at different scales. Cai et al. [17] proposed TB-FPN, which enhances tooth segmentation through a cascade boundary-aware feature pyramid network and improves the representation of multi-scale boundary information.

Second, Transformer-based and hybrid architectures have been explored to strengthen global context modeling. Li et al. [18] proposed a diffusion-based Conv-Former dual-encoder U-Net for the MICCAI STS-2D challenge, combining convolutional structures with advanced global modeling strategies. Wang et al. [14] summarized semi-supervised approaches for the STS challenge, including Transformer-based segmentation variants such as STS-TransU-Net. Hao et al. [22] proposed a semi-supervised Transformer-based framework for automated tooth segmentation and identification on panoramic radiographs, showing the potential of Transformer architectures for exploiting unlabeled data and modeling global tooth arrangement. Wang et al. [23] further reported the MICCAI STS 2024 Challenge, which extends tooth analysis toward semi-supervised instance-level segmentation in both panoramic X-ray and CBCT images. These methods improve global feature interaction to some extent, but their boundary refinement ability still depends heavily on the decoding process.

Third, several methods focus on edge-related information, tooth numbering, or post-processing strategies. Leng et al. [19] proposed UX-CNet to improve tooth segmentation by enhancing edge information acquisition. Lin et al. [20] introduced a self-training U-Net with a denoiser for semi-supervised tooth segmentation in X-ray images. In addition, Wang et al. [21] improved segmentation results through special post-processing after tooth segmentation. Wang et al. [24] proposed STSN-Net to simultaneously perform tooth segmentation and numbering in crowded environments, indicating that tooth separation and structural identification are closely coupled in complex dental scenes. Zhang et al. [25] proposed a boundary feature fusion network for tooth image segmentation, demonstrating the usefulness of boundary feature fusion for improving tooth contour delineation.

Although these methods have achieved promising results, most of them mainly focus on improving region-level accuracy, multi-scale feature fusion, semi-supervised learning, or post-processing strategies. Precise boundary modeling remains insufficiently addressed, especially in challenging cases involving tooth adhesion, low contrast, and blurred boundaries. In addition, methods that emphasize global context modeling do not necessarily guarantee accurate local boundary delineation, while edge-aware methods may still lack sufficient modeling of the global dental arch structure. Therefore, panoramic dental X-ray segmentation requires a unified framework that can simultaneously enhance spatial structural representation and explicit boundary refinement.

Considering these challenges, a suitable baseline for dental X-ray segmentation should satisfy three requirements: stable encoder–decoder feature reconstruction, sufficient local detail preservation, and convenient integration with attention and boundary-aware modules. ResU-Net meets these requirements by combining the multi-scale skip-connection structure of U-Net with residual learning, which improves feature propagation and training stability. Therefore, instead of adopting a more complex Transformer-based framework as the baseline, this study selects ResU-Net as the backbone to provide a simple, stable, and extensible foundation for further incorporating coordinate attention and explicit boundary modeling.

### 2.2. ResU-Net and Its Variants

As discussed above, panoramic dental X-ray segmentation requires a backbone that can preserve local details while maintaining stable feature reconstruction. The U-Net architecture is a classical encoder–decoder backbone for medical image segmentation, and numerous studies have extended it to enhance feature representation capability. Residual learning was originally proposed to alleviate the degradation problem in deep neural networks and improve feature propagation [26]. Based on this idea, residual structures have been widely incorporated into U-Net-like segmentation frameworks.

For instance, R2U-Net [27] combines residual connections with recurrent convolutional layers to replace the original U-Net sub-modules, preserving network depth while mitigating gradient degradation and demonstrating strong capability in extracting low-level features. ResUNet++ [28] further enhances feature representation by integrating residual blocks with dense connections and multi-scale feature fusion. AnatomyNet [29] replaces traditional convolutional units with residual blocks integrated with Squeeze-and-Excitation attention, enabling effective multi-scale feature learning for small anatomical structures.

Overall, U-Net variants based on residual structures have become mainstream backbone networks in medical image segmentation, providing a solid foundation for performance improvement. Therefore, ResU-Net is adopted in this work as the backbone due to its balance between representation capability and training stability.

However, for dental X-ray images characterized by low contrast, blurred boundaries, and narrow inter-tooth gaps, ResU-Net alone still struggles to accurately separate adjacent teeth and preserve fine boundary details. In particular, convolution-based models mainly rely on local receptive fields, which may lead to insufficient modeling of the global dental arch structure and ambiguous separation of adjacent teeth [30]. These limitations indicate that improving spatial awareness and introducing explicit boundary constraints are essential for accurate dental X-ray segmentation, which motivates the design of the proposed method.

### 2.3. Attention Mechanisms

Attention mechanisms have been widely applied in medical image segmentation to adaptively reweight feature importance and enhance the representation of target regions. Early approaches, such as the Squeeze-and-Excitation (SE) module [31], model inter-channel dependencies to recalibrate feature responses, while the Convolutional Block Attention Module (CBAM) [32] further integrates both channel and spatial attention to refine feature maps along two dimensions. Non-local neural networks [33] further demonstrate that capturing long-range dependencies can improve global contextual representation. However, these methods mainly focus on channel importance, local spatial regions, or general global dependencies, and lack the ability to explicitly encode precise positional information.

To address this limitation, CA has been proposed, which embeds direction-aware spatial encoding into channel attention, enabling the model to capture both long-range dependencies and accurate positional information [12]. Specifically, CA decomposes global pooling into horizontal and vertical directions, allowing the network to preserve positional information while modeling channel relationships.

For dental image segmentation tasks, the tooth arrangement exhibits strong spatial regularity, and the spacing between adjacent teeth is relatively small, which imposes high demands on precise positional perception. By encoding positional information along horizontal and vertical directions, CA enables the network to simultaneously capture the global arrangement of the dental arch and local structural details. To this end, we employ CA as a lightweight coordinate-aware feature enhancement component, which is further combined with explicit boundary extraction and decoder-level boundary injection to address the specific challenge of panoramic tooth separation. As a result, it can effectively model the spatial distribution and boundary location of teeth, thereby improving the discrimination between adjacent teeth and enhancing the morphological consistency of segmentation results.

Nevertheless, attention mechanisms mainly enhance feature representation and spatial perception, but they do not explicitly model tooth boundaries. Therefore, relying only on attention may still be insufficient in cases with blurred contours, weak contrast, or adhesive inter-tooth regions. This limitation suggests that coordinate-aware spatial modeling should be combined with explicit boundary refinement to further improve tooth segmentation performance.

### 2.4. Boundary Modeling Methods

In medical image segmentation, boundary information plays a critical role in accurately delineating target regions. However, existing deep learning models often neglect explicit boundary learning. Previous studies have shown that introducing boundary supervision or edge detection operators can improve the model’s ability to identify object boundaries [34]. To this end, many approaches adopt multi-task learning strategies that jointly optimize boundary detection and segmentation, or introduce dedicated edge branches to strengthen boundary features.

For example, DCAN [35] introduces contour-aware learning to jointly model object regions and contours, improving the delineation of gland boundaries. Boundary loss further demonstrates that boundary-aware optimization can improve segmentation performance, especially in highly unbalanced segmentation tasks [36]. Gated-SCNN [37] introduces a shape stream to enhance boundary-sensitive feature representation for semantic segmentation, while BASNet [38] incorporates boundary-aware learning to improve object contour localization. More recently, Lin et al. [13] proposed a systematic boundary modeling framework and introduced BEM and BIM, which extract gradient-based boundary information and inject boundary cues into the decoder for improved segmentation refinement. In the dental imaging field, Zhang et al. [25] introduced a boundary feature fusion network to enhance tooth image segmentation by fusing boundary-related features, further confirming the importance of boundary cues for dental contour delineation.

Boundary-aware modeling strategies have emerged as important research directions in medical image segmentation. Particularly for dental X-ray segmentation tasks, due to the low grayscale contrast between teeth and alveolar bone, as well as the narrow inter-tooth gaps that are highly susceptible to noise, traditional methods often struggle to accurately delineate tooth contours and effectively separate adjacent structures. Therefore, introducing explicit boundary modeling strategies is both necessary and highly task-relevant.

However, boundary modeling alone mainly focuses on local contour refinement and may not fully capture the global spatial arrangement of the dentition. In panoramic dental X-ray images, accurate segmentation requires both global structural perception and local boundary discrimination. Notably, the encoder–decoder structure of ResU-Net naturally accommodates the injection of boundary priors, making the combination of ResU-Net with CA and BEM/BIM a promising solution for reducing tooth adhesion and improving boundary precision.

Different from methods that only add boundary supervision at the final output, use an auxiliary edge branch, or fuse boundary features as an independent refinement component, the proposed framework extracts multi-level boundary priors by BEM and progressively injects them into the decoding process through BIM. This design is intended to enhance tooth contours while suppressing ambiguous background responses in narrow inter-tooth gaps.

## 3. Methodology

### 3.1. Overall Architecture

**Figure 2:**
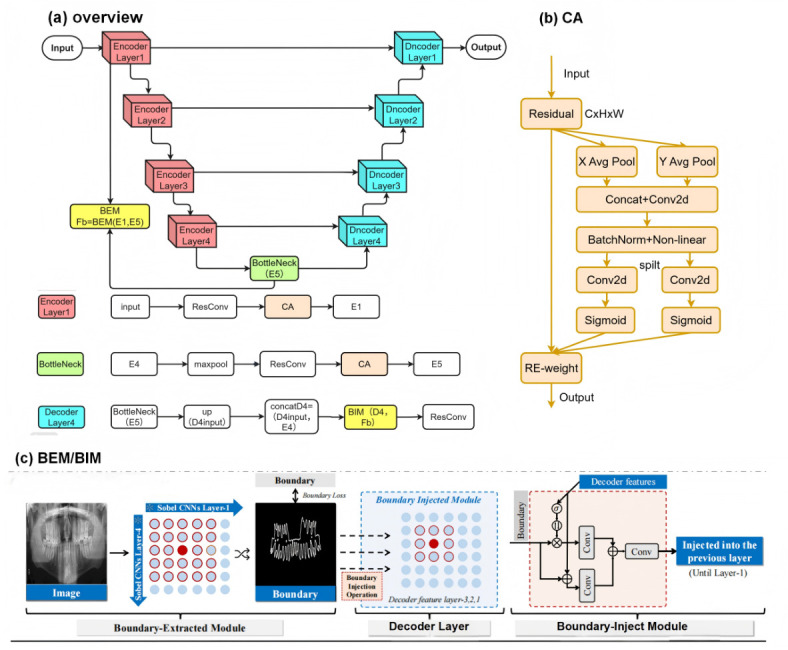
Architecture of BA-ResUNet. (**a**) Overall encoder–decoder framework with CA and BEM/BIM. (**b**) Coordinate Attention module. (**c**) Boundary extraction and injection module.

In this work, we propose a tooth X-ray image segmentation framework based on ResU-Net. As illustrated in Figure 2a, the framework follows an encoder–decoder structure with skip connections. The encoder is composed of residual convolutional blocks, where a CA module is embedded at the end of each block to capture global spatial dependencies by decoupling horizontal and vertical coordinate information. The BEM operates on both the bottleneck and high-level features. It takes shallow and deep features as input and employs Sobel operators to extract horizontal and vertical gradient information, thereby generating boundary-enhanced features [13,34].

In the decoder, BIM is introduced to inject explicit boundary information into the segmentation process through a dual-path fusion of the previous decoding features and the boundary features generated by BEM. The entire network is trained end-to-end with a composite loss that jointly optimizes the main segmentation task and the auxiliary boundary detection task. This design is simple yet effective, combining lightweight spatial attention with explicit boundary modeling, and significantly improves sensitivity to boundary details.

### 3.2. Coordinate Attention Module (CA)

To strengthen the model’s ability to capture the overall arrangement of the dental arch and preserve spatial positional information, we embed a CA module [12] after each residual convolutional block in the encoder of ResU-Net, as illustrated in Figure 2b.

Let the output feature map of the residual convolution block at the *i*-th encoder stage be a three-dimensional tensor with dimensions $C_{i}\times H_{i}\times W_{i}$, where $C_{i}$, $H_{i}$ and $W_{i}$ denote the number of channels, height, and width, respectively. The CA module first performs one-dimensional global average pooling along the horizontal and vertical directions separately. This operation yields two direction-specific feature descriptors, one encoding height-related information and the other encoding width-related information. Such direction-decomposed pooling can capture long-range dependencies along one spatial axis while preserving accurate positional information along the other axis. Then, the two directional descriptors are concatenated and passed through a 1×1 convolution for channel compression, followed by a nonlinear activation function to produce an intermediate representation. The compressed feature is then split into two branches corresponding to the horizontal and vertical directions, and each branch is transformed through another 1×1 convolution to generate a direction-aware attention map. Finally, these two attention maps are element-wise multiplied with the original feature map to perform spatial recalibration. The resulting output feature preserves global contextual information while becoming more sensitive to location.

From a structural perspective, the CA module is inserted after each encoder residual convolution block and before down-sampling. This design introduces only a small number of additional parameters with negligible computational overhead, while significantly improving the model’s ability to perceive the spatial arrangement and relative positional relationships of teeth.

### 3.3. Boundary Extraction and Boundary Injection

In dental X-ray images, neighboring teeth often exhibit very low grayscale contrast, and the inter-tooth gaps are typically narrow. As a result, the decoder is prone to foreground over-expansion, which leads to adhesion between adjacent teeth. To address this issue, we introduce an explicit boundary modeling mechanism, including the BEM and BIM. Their detailed structures are illustrated in Figure 2c.

#### 3.3.1. Boundary Extraction Module (BEM)

The BEM extracts a compact boundary prior through Sobel filtering and multi-scale feature fusion. Specifically, for an input feature map *F*, Sobel filtering is used to obtain a boundary-sensitive response:

(1) $$S\left(F\right)=\sqrt{{\left(K_{x}*F\right)}^{2}+{\left(K_{y}*F\right)}^{2}},$$

where $K_{x}$ and $K_{y}$ are the horizontal and vertical Sobel kernels [39], respectively. The shallow response $S(E_{1})$ and the up-sampled bottleneck response $S(E_{5})$ are then fused by convolution to generate the boundary prior feature $F_{b}$:

(2) $$F_{b}=\psi_{b}\left([E_{1},\mathrm{Up}\left(E_{5}\right),S\left(E_{1}\right),\mathrm{Up}\left(S\left(E_{5}\right)\right)]\right),$$

where [·] denotes channel-wise concatenation, Up(·) denotes bilinear up-sampling, and $\psi_{b}\left(\cdot \right)$ is a convolutional fusion block. During training, $F_{b}$ is supervised by a boundary ground-truth mask $B_{\mathrm{gt}}$ derived from the segmentation annotation *M*. Specifically, $B_{\mathrm{gt}}$ is generated by a morphological gradient operation, which is computed as the difference between the dilated and eroded segmentation masks:

(3) $$B_{\mathrm{gt}}=\mathrm{MaxPool}\left(M\right)-\mathrm{MinPool}\left(M\right),$$

where MaxPool(·) and MinPool(·) denote the dilation and erosion operations, respectively, and *M* denotes the ground-truth segmentation mask. In this work, the morphological operation is implemented using a 3×3 kernel. This kernel size considers the immediate 8-neighborhood of each pixel and extracts a thin boundary band around tooth contours, which is suitable for providing localized boundary supervision without introducing excessively thick boundary regions. This strategy provides a simple yet effective form of boundary supervision while alleviating the class imbalance problem at boundary regions.

#### 3.3.2. Boundary Injection Module (BIM)

The BIM progressively injects the boundary-enhanced feature $F_{b}$ into each decoder stage to guide boundary-aware reconstruction. For the *j*-th decoder stage, the inputs include the skip-connected encoder feature $E_{j}$, the decoder feature $D_{j-1}$ from the previous stage, and $F_{b}$ generated by BEM. Before fusion, $D_{j-1}$ and $F_{b}$ are resized by bilinear interpolation to the spatial resolution of $E_{j}$, and convolutional layers are used to align the channel dimensions.

BIM contains two complementary paths. The foreground enhancement path concatenates the aligned encoder, decoder, and boundary features to strengthen tooth contour responses:

(4) $$F_{fg}^{j}=\psi_{fg}^{j}\left(\left[E_{j},\mathrm{Up}\left(D_{j-1}\right),\mathrm{Up}\left(F_{b}\right)\right]\right),$$

where [·] denotes channel-wise concatenation and $\psi_{fg}^{j}\left(\cdot \right)$ denotes a convolutional fusion block. In parallel, the background suppression path generates a background attention map from the decoder response and suppresses ambiguous non-tooth regions:

(5) $$A_{bg}^{j}=1-\sigma \left({\mathrm{Mean}}_{c}\left(\mathrm{Up}\left(D_{j-1}\right)\right)\right),$$

(6) $$F_{bg}^{j}=\psi_{bg}^{j}\left(E_{j}⊙A_{bg}^{j}\right),$$

where ${\mathrm{Mean}}_{c}\left(\cdot \right)$ denotes channel-wise averaging and ⊙ denotes element-wise multiplication. Finally, the current decoder output is obtained by fusing the foreground-enhanced feature, the background-suppressed feature, and the original decoder feature:

(7) $$D_{j}=\psi_{out}^{j}\left(\left[F_{fg}^{j},F_{bg}^{j},\mathrm{Up}\left(D_{j-1}\right)\right]\right).$$

By preserving the original decoder feature in the final fusion, BIM forms an information-preserving residual-like shortcut that maintains feature continuity across decoder stages. Meanwhile, the progressive injection of multi-level boundary priors provides explicit contour guidance during decoding. This design helps enhance tooth boundaries, suppress foreground over-expansion, and preserve narrow inter-tooth gaps.

### 3.4. Loss Function

A composite loss is used to train the network, including a segmentation loss $L_{\mathrm{seg}}$ and a boundary loss $L_{\mathrm{bnd}}$. The total loss function is defined as follows:

(8) $$L_{\mathrm{total}}=L_{\mathrm{seg}}+\lambda L_{\mathrm{bnd}},$$

where λ is the boundary loss weight coefficient used to balance the segmentation loss and the boundary loss. In this study, λ is set to 3, and the parameter sensitivity analysis is presented in Section 4.3.

The segmentation loss adopts a BCE–Dice [40] form:

(9) $$L_{\mathrm{seg}}=0.5L_{\mathrm{BCE}}+0.5L_{\mathrm{Dice}},$$

(10) $$L_{\mathrm{BCE}}=-\frac{1}{N}\sum_{i=1}^{N}\left[y_{i}\log\left({\hat{y}}_{i}\right)+\left(1-y_{i}\right)\log\left(1-{\hat{y}}_{i}\right)\right],$$

(11) $$L_{\mathrm{Dice}}=1-\frac{2|X\cap Y|+\varepsilon }{|X|+|Y|+\varepsilon },$$

where $y_{i}$ represents the ground-truth label, ${\hat{y}}_{i}$ denotes the predicted probability, and *N* is the total number of pixels. *X* represents the set of predicted pixels, *Y* denotes the ground-truth set, and ε is a smoothing term.

The boundary loss is defined as follows:

(12) $$L_{\mathrm{bnd}}=L_{\mathrm{Dice}}\left(B_{\mathrm{pred}},B_{\mathrm{gt}}\right).$$

The boundary loss is computed using the Dice loss to measure the discrepancy between the predicted boundary map $B_{\mathrm{pred}}$ and the ground-truth boundary map $B_{\mathrm{gt}}$ defined in Section 3.3.1. This formulation provides an efficient way to generate supervision signals directly from the segmentation mask, enabling the model to focus on boundary regions during training.

## 4. Experiments and Results

### 4.1. Experimental Settings

#### 4.1.1. Datasets

The performance of the proposed model was mainly evaluated on the public MICCAI STS-2023 dental dataset [14]. This dataset contains panoramic dental images collected from Hangzhou Dental Group, Hangzhou Qiantang Stomatological Hospital, the University of Electronic Science and Technology of China, and Queen Mary University of London. And it provides an official partition of 2000 images for training and 500 for testing, which has been widely adopted in prior works to ensure fair comparison. To further assess the generalization capability of the proposed model, additional experiments were performed on other publicly available datasets, including MICCAI STS-2024 [23], Hand-bone X-ray [41], Chest-bone X-ray [42] and Fracture-region X-ray [43], covering different anatomical structures. The splits of these datasets also follow their official partitions.

#### 4.1.2. Implementation Details

Our method is implemented with Python 3.8 and PyTorch 1.11.0 and trained on a single NVIDIA RTX 4090D (24 GB) GPU. The main training parameters are summarized in Table 1.

#### 4.1.3. Evaluation Metrics

In this study, the Dice coefficient is adopted as the primary evaluation metric to measure the overlap between the predicted results and the ground truth. The Intersection over Union (IoU) is used to evaluate the region-level intersection performance. In addition, the Hausdorff Distance (HD) is introduced to quantify the maximum deviation between the predicted boundary and the ground-truth boundary, providing a more sensitive assessment of boundary quality [44]. These metrics are defined as:

(13) $$\mathrm{Dice}=\frac{2|X\cap Y|}{\left|X|+|Y\right|},$$

(14) $$\mathrm{IoU}=\frac{\left|X\cap Y\right|}{\left|X\cup Y\right|},$$

(15) $$\mathrm{HD}\left(X,Y\right)=\max\left\{h(X,Y),h(Y,X)\right\},$$

(16) $$\begin{array}{cc} h(X,Y) & =\max_{x\in X}\min_{y\in Y}d\left(x,y\right),\\ h(Y,X) & =\max_{y\in Y}\min_{x\in X}d\left(y,x\right), \end{array}$$

where *X* and *Y* denote the sets of predicted results and ground-truth labels, and d(x,y) represents the Euclidean distance between points *x* and *y*.

### 4.2. Comparison with Other Methods

To evaluate the effectiveness of the proposed BA-ResUNet, we compare it with representative methods from the MICCAI STS-2D challenge and recent literature. As discussed in Section 2.1, these methods cover different technical strategies, including data augmentation, semi-supervised learning, boundary-aware design, edge-sensitive modeling, Transformer-based hybrid architectures, multi-scale CNNs, and post-processing strategies.

As summarized in Table 2, The proposed BA-ResUNet achieves the highest Dice and IoU values among all compared methods, reaching 94.46% and 98.61%, respectively. Since Dice and IoU mainly measure the overlap between predicted regions and ground-truth regions, these results indicate that the proposed method achieves the best region-level segmentation performance among the compared methods. In terms of HD, the proposed method obtains a value of 0.0238, which is the second-best result and is close to the best value of 0.0230 reported by Wang et al. [21]. Since HD is sensitive to the maximum boundary deviation between the predicted boundary and the ground-truth boundary, this result suggests that the proposed method maintains competitive boundary localization performance. However, as the HD value is still slightly higher than the best reported result, further improvement is still possible in reducing extreme boundary-point deviations.

Overall, the comparison demonstrates that BA-ResUNet achieves strong region-level segmentation accuracy while maintaining competitive boundary performance. These results suggest that integrating coordinate-aware spatial representation with explicit boundary modeling is effective for panoramic tooth segmentation. More detailed analysis of the contributions of CA and BEM/BIM is provided in the ablation study and qualitative analysis in Section 4.3.

### 4.3. Generalization Evaluation on Additional X-Ray Datasets

To assess generalization beyond tooth segmentation, we conducted additional experiments on several public X-ray datasets. These include other dental panoramic datasets: MICCAI STS-2024, Hand-bone X-ray, Chest X-ray and Fracture-region X-ray. For each dataset, the baseline and the BA-ResUNet were trained and evaluated under the same dataset protocol to ensure a fair comparison. The results are summarized in Table 3.

As shown in Table 3, BA-ResUNet consistently outperforms the baseline across all four datasets in terms of Dice, IoU, and HD. For the MICCAI STS-2024 dataset, BA-ResUNet improves Dice by 0.29% (from 95.02% to 95.31%) and IoU by 0.79% (from 98.14% to 98.93%). Similar gains are observed on the other three datasets. These consistent improvements indicate that the proposed coordinate-aware feature enhancement and boundary-guided decoding are not only effective for tooth segmentation but also transferable to other X-ray segmentation tasks where object boundaries are weak and adjacent anatomical structures are difficult to separate.

### 4.4. Ablation Experiments

#### 4.4.1. Parameter Sensitivity of Boundary Loss Weight

Before conducting the component ablation experiments, the influence of the boundary loss weight λ was first evaluated on the validation set. As shown in Table 4, different values of λ lead to different balances between the main segmentation objective and the auxiliary boundary supervision. When λ is set to 1, the contribution of boundary supervision is relatively weak, resulting in lower Dice and IoU values and a larger HD. As λ increases to 3, the model achieves the best overall performance, with the highest Dice and IoU values and the lowest HD. However, when λ is further increased to 4, the Dice and IoU slightly decrease, indicating that excessive boundary supervision may interfere with the main segmentation task. Therefore, λ is set to 3 in all subsequent experiments.

#### 4.4.2. Component Ablation Study

To evaluate the contribution of each proposed component and further analyze the error patterns of the model, quantitative and qualitative ablation experiments were conducted. The quantitative results are summarized in Table 5. In this experiment, each metric is reported as mean ± standard deviation, and two-sided paired Wilcoxon signed-rank tests were performed at the image-level between the baseline and each enhanced variant. The baseline already achieves a Dice of 94.15% and an IoU of 96.18%. After introducing CA alone, the Dice increases from 94.15% to 94.33% (p<0.001), the IoU increases from 96.18% to 97.71% (p<0.001). These results indicate that coordinate attention improves the spatial representation ability of the encoder and helps the network capture the global arrangement of teeth more effectively. When BEM/BIM modules are introduced without CA, the Dice reaches 94.30%, the IoU increases to 98.03%, and the HD decreases to 0.0284. Compared with CA alone, BEM/BIM produce more improvement in IoU and a greater reduction in HD, suggesting that explicit boundary extraction and boundary-guided injection contribute more directly to improving region overlap and boundary localization in narrow inter-tooth regions. However, the Dice improvement does not reach statistical significance, indicating that boundary modules alone do not consistently enhance overall region overlap across all samples. When CA and BEM/BIM are integrated into the baseline, BA-ResUNet achieves the best overall performance, with a Dice of 94.46% (an increase of 0.31% over the baseline, p<0.001), an IoU of 98.61% (an increase of 2.43%, p<0.001), and an HD of 0.0238 (a reduction of 0.0087, p<0.05). The joint integration of CA and BEM/BIM yields statistically significant improvements in all three metrics, demonstrating that coordinate attention and explicit boundary modeling play complementary roles: CA strengthens spatial and region-level representation, while BEM/BIM provides complementary boundary guidance during decoding. Their combination therefore improves both segmentation overlap and boundary localization more consistently than either component alone.

**Figure 3:**
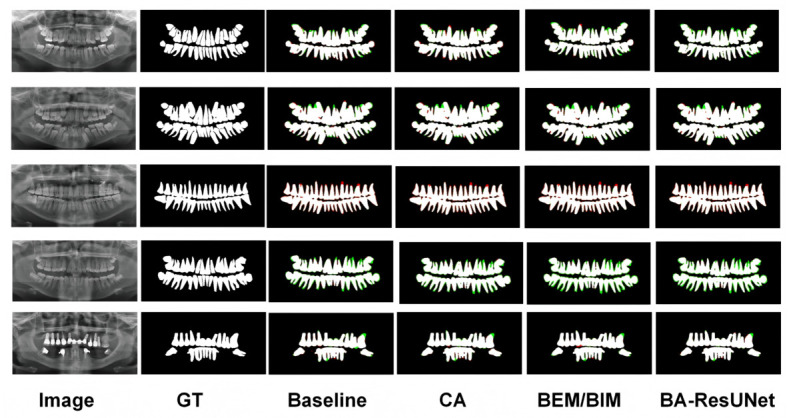
Qualitative ablation comparison of different model variants on panoramic dental X-ray images.

The qualitative ablation comparison in Figure 3 further supports these findings. In Figure 3, white regions indicate correctly segmented tooth areas, red regions denote over-segmentation errors, and green regions denote under-segmentation errors. Compared with the baseline, the introduction of CA leads to more consistent tooth structures, suggesting that coordinate attention is beneficial for enhancing spatial representation and structural coherence. After incorporating BEM/BIM, the predicted contours become closer to the ground truth, especially in local boundary regions, which is consistent with the boundary-oriented design of these modules. When CA and BEM/BIM are jointly introduced, the complete model produces the visually best results across most samples. The progressive improvements shown in Table 5 and Figure 3 suggest that CA and BEM/BIM contribute differently but complementarily to the final segmentation performance.

**Figure 4:**
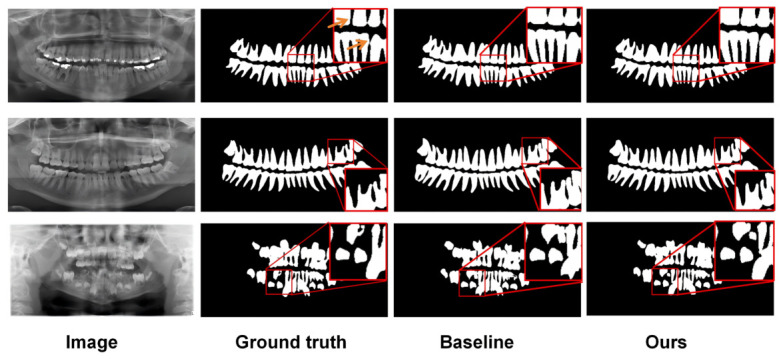
Qualitative comparison between the baseline and the proposed method on challenging cases.

To further investigate how these improvements are reflected in representative difficult cases, qualitative comparisons were conducted on challenging samples together with the statistics of over-segmentation and under-segmentation ratios. Figure 4 presents three representative difficulties in panoramic tooth segmentation, namely tooth adhesion, low contrast, and blurred boundaries. Specifically, the first row presents a typical adhesion case, where adjacent teeth are closely attached and difficult to separate. The second row corresponds to a low-contrast case, in which the grayscale difference between teeth and surrounding tissues is weak. The third row illustrates a blurred-boundary case, where the tooth contours are indistinct and difficult to localize precisely. In all three scenarios, the proposed model produces predictions that are visually closer to the ground truth than the baseline. In the enlarged local regions marked by the red boxes, the baseline exhibits more obvious contour deviation, including local over-segmentation or incomplete delineation, whereas the proposed model achieves better local fitting and more complete structural recovery. These visual results indicate that the proposed framework is more robust when dealing with the common challenges of panoramic tooth segmentation.

**Figure 5:**
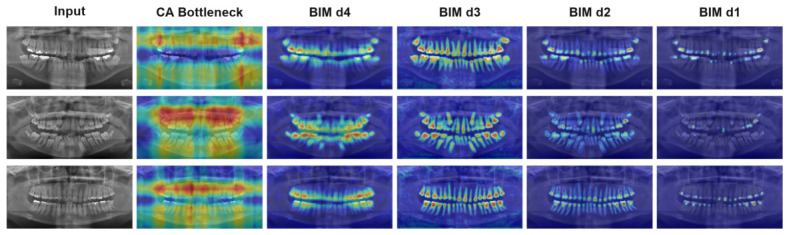
Visualization of CA bottleneck response and progressive BIM decoder responses on representative panoramic dental X-ray images.

To provide more intuitive evidence for the effects of CA and BEM/BIM, Figure 5 visualizes the CA bottleneck response and the progressive BIM decoder responses. The CA bottleneck map highlights the overall tooth-bearing region and dental-arch structure, indicating that coordinate attention helps preserve global spatial organization. From BIM d4 to BIM d1, the decoder responses gradually shift from coarse dental-arch activation to finer boundary-related responses, showing that the boundary prior is progressively injected into the decoding process rather than being used only at the final output stage.

This observation is also supported by the error-ratio statistics in Table 6. Compared with the baseline, the proposed model reduces the mean over-segmentation ratio from 6.02% to 5.55%, while the mean under-segmentation ratio decreases from 5.78% to 5.63%. Meanwhile, the Dice mean increases from 94.12% to 94.46%, and the standard deviations remain comparable. These results suggest that the proposed model not only improves the average segmentation accuracy, but also maintains relatively stable performance across the validation samples. Taken together, the quantitative improvements in Table 2, the qualitative comparisons in Figure 3 and Figure 4, and the error-ratio analysis in Table 6 consistently demonstrate that the proposed method improves both regional overlap and boundary localization performance. More importantly, the results on representative adhesion, low-contrast, and blurred-boundary samples provide intuitive evidence that the proposed framework is better suited for handling the common challenges in panoramic tooth segmentation.

## 5. Conclusions

To address common challenges in panoramic dental X-ray images, including tooth adhesion, low contrast, and blurred boundaries, we propose a boundary-aware dental X-ray segmentation framework based on ResU-Net, integrating CA with explicit boundary modeling through the BEM and BIM. By combining spatial feature enhancement with structured boundary guidance during decoding, the proposed framework effectively improves inter-tooth separation and boundary delineation. Experimental results on the MICCAI STS-2D dataset demonstrate consistent improvements in Dice and IoU, indicating enhanced segmentation accuracy and robustness while maintaining a lightweight and practical architecture. Additional evaluations on multiple public X-ray datasets further indicate the generalization potential of the proposed boundary-guided design.

Future work will focus on exploring more efficient boundary modeling strategies, lightweight network designs, and further dental-specific external validation to improve the generalization capability of the proposed method.

## Figures and Tables

**Table 1:** Training parameters.

Parameter	Value
Batch size	8
Total epochs	150
Optimizer	AdamW
Initial learning rate	$1\times 10^{-4}$
Weight decay	$5\times 10^{-4}$
Learning rate scheduler	Cosine Annealing LR

**Table 2:** Comparison with representative methods on the STS-2D dataset.

Method	Dice (%) ↑	IoU (%) ↑	HD (mm) ↓
Shen et al. (2025) [15]	89.02	96.70	0.0368
Cai et al. (2025) [17]	91.75	97.70	0.0250
Li et al (2025) [18]	91.81	96.35	0.0332
Lin et al. (2025) [20]	93.15	98.03	0.0232
Wang et al. (2026) [14]	93.18	96.91	0.0298
Wang et al. (2025) [16]	93.41	98.14	0.0244
Leng et al. (2025) [19]	93.62	96.98	0.0300
Wang et al. (2025) [21]	94.28	98.54	**0.0230**
BA-ResUNet (Ours)	**94.46**	**98.61**	0.0238

↑ indicates that higher values are better; ↓ indicates that lower values are better. Bold values denote the best performance.

**Table 3:** Generalization results on different X-ray datasets.

Dataset	Dice (%) ↑		IoU (%) ↑		HD (mm) ↓	
	Baseline	Ours	Baseline	Ours	Baseline	Ours
MICCAI STS 2024 [23]	95.02	**95.31**	98.14	**98.93**	0.0287	**0.0209**
Hand-bone X-ray [41]	95.38	**95.67**	98.22	**99.01**	0.0279	**0.0204**
Chest X-ray [42]	94.82	**95.16**	98.05	**98.88**	0.0294	**0.0213**
Fracture-region X-ray [43]	92.71	**92.94**	94.36	**96.12**	0.0338	**0.0247**

↑ indicates that higher values are better; ↓ indicates that lower values are better. Bold values denote the best performance.

**Table 4:** Parameter sensitivity analysis of λ on the validation set. The best results are highlighted in bold.

λ	Dice (%) ↑	IoU (%) ↑	HD (mm) ↓
1	93.98	97.56	0.0284
2	94.25	98.01	0.0259
3	**94.41**	**98.61**	**0.0238**
4	94.23	98.05	0.0257

↑ indicates that higher values are better; ↓ indicates that lower values are better.

**Table 5:** Ablation results with statistical significance analysis.

Method	CA	BEM/BIM	Dice (%) ↑	IoU (%) ↑	HD (mm) ↓
ResU-Net (Baseline)	×	×	94.15±3.49	96.18±0.59	0.0325±0.0051
CA	✓	×	94.33±3.46 ***	97.71±0.58 ***	0.0298±0.0062
BEM/BIM	×	✓	94.30±3.47	98.03±0.61 *	0.0284±0.0072
BA-ResUNet (Ours)	✓	✓	94.46±3.46 ***	98.61±0.61 ***	0.0238±0.0064 *

* and *** indicate p<0.05 and p<0.001, respectively.

**Table 6:** Comparison of Dice and error-ratio statistics on the validation set. The best results are highlighted in bold.

Model	Dice (Mean ± Std) (%)	Over-Seg Ratio (%)	Under-Seg Ratio (%)
Baseline	94.15 ± 3.49	6.02	5.78
CA	94.33 ± 3.46	5.62	5.65
BEM/BIM	94.30 ± 3.47	5.86	5.72
BA-ResUNet	**94.46 ± 3.46**	**5.55**	**5.63**

## Data Availability

The MICCAI STS-2D dataset is available from the official STS Challenge page: https://tianchi.aliyun.com/dataset/156596 (accessed on 1 July 2025).
